# 5-Ethyl-3-(2-fluoro­phenyl­sulfon­yl)-2-methyl-1-benzofuran

**DOI:** 10.1107/S1600536812036872

**Published:** 2012-09-05

**Authors:** Hong Dae Choi, Pil Ja Seo, Uk Lee

**Affiliations:** a Department of Chemistry, Dongeui University, San 24 Kaya-dong, Busanjin-gu, Busan 614-714, Republic of Korea; b Department of Chemistry, Pukyong National University, 599-1 Daeyeon 3-dong, Nam-gu, Busan 608-737, Republic of Korea

## Abstract

In the title compound, C_17_H_15_FO_3_S, the 2-fluoro­phenyl ring makes a dihedral angle of 89.12 (8)° with the mean plane of the benzofuran fragment. In the crystal, mol­ecules are linked by weak C—H⋯O and C—H⋯π inter­actions.

## Related literature

For background information and the crystal structures of related compounds, see: Choi *et al.* (2010 ▶, 2011 ▶).
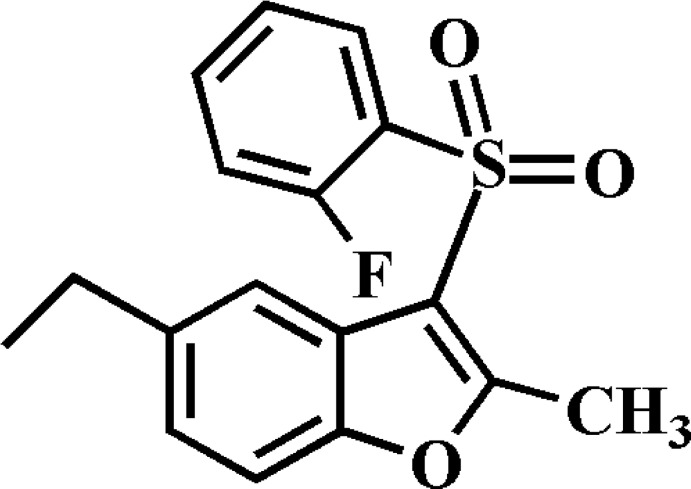


## Experimental

### 

#### Crystal data

C_17_H_15_FO_3_S
*M* *_r_* = 318.35
Monoclinic, 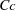

*a* = 11.290 (2) Å
*b* = 16.171 (3) Å
*c* = 8.5612 (14) Å
β = 105.045 (11)°
*V* = 1509.4 (5) Å^3^
*Z* = 4
Mo *K*α radiation
μ = 0.24 mm^−1^
*T* = 173 K
0.38 × 0.30 × 0.27 mm

#### Data collection

Bruker SMART APEXII CCD diffractometer
Absorption correction: multi-scan (*SADABS*; Bruker, 2009 ▶) *T*_min_ = 0.545, *T*_max_ = 0.746
7108 measured reflections
3129 independent reflections
2676 reflections with *I* > 2σ(*I*)
*R*_int_ = 0.035

#### Refinement

*R*[*F* ^2^ > 2σ(*F* ^2^)] = 0.042
*wR*(*F* ^2^) = 0.105
*S* = 1.06
3129 reflections
201 parameters
2 restraints
H-atom parameters constrained
Δρ_max_ = 0.61 e Å^−3^
Δρ_min_ = −0.35 e Å^−3^
Absolute structure: Flack (1983 ▶), 1271 Friedel pairs
Flack parameter: −0.05 (8)

### 

Data collection: *APEX2* (Bruker, 2009 ▶); cell refinement: *SAINT* (Bruker, 2009 ▶); data reduction: *SAINT*; program(s) used to solve structure: *SHELXS97* (Sheldrick, 2008 ▶); program(s) used to refine structure: *SHELXL97* (Sheldrick, 2008 ▶); molecular graphics: *ORTEP-3* (Farrugia, 2012 ▶) and *DIAMOND* (Brandenburg, 1998 ▶); software used to prepare material for publication: *SHELXL97*.

## Supplementary Material

Crystal structure: contains datablock(s) global, I. DOI: 10.1107/S1600536812036872/fj2589sup1.cif

Structure factors: contains datablock(s) I. DOI: 10.1107/S1600536812036872/fj2589Isup2.hkl

Supplementary material file. DOI: 10.1107/S1600536812036872/fj2589Isup3.cml

Additional supplementary materials:  crystallographic information; 3D view; checkCIF report

## Figures and Tables

**Table 1:** Hydrogen-bond geometry (Å, °) *Cg* is the centroid of the C12–C17 2-fluoro­phenyl ring.

*D*—H⋯*A*	*D*—H	H⋯*A*	*D*⋯*A*	*D*—H⋯*A*
C15—H15⋯O3^i^	0.95	2.53	3.420 (3)	156
C16—H16⋯O2^ii^	0.95	2.49	3.121 (4)	124
C5—H5⋯*Cg* ^iii^	0.95	2.79	3.692 (3)	159

Symmetry codes: (i) 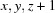; (ii) 
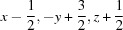
; (iii) 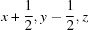.

**Fig. 1.**
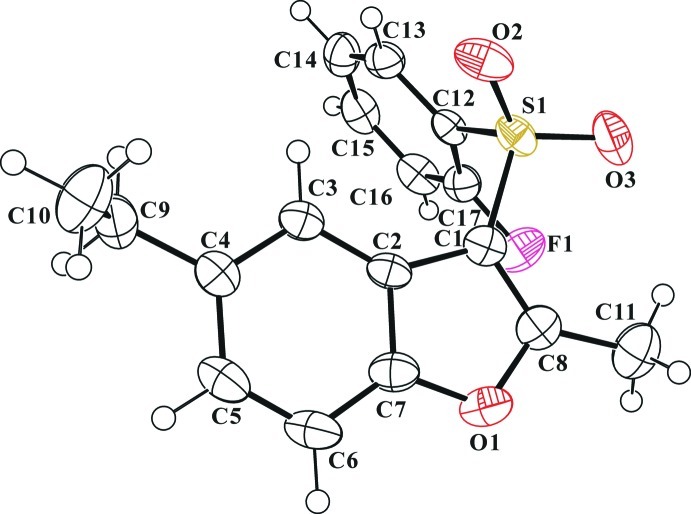
The molecular structure of the title compound with the atom numbering scheme. Displacement ellipsoids are drawn at the 50% probability level. H atoms are presented as small spheres of arbitrary radius.

**Fig. 2.**
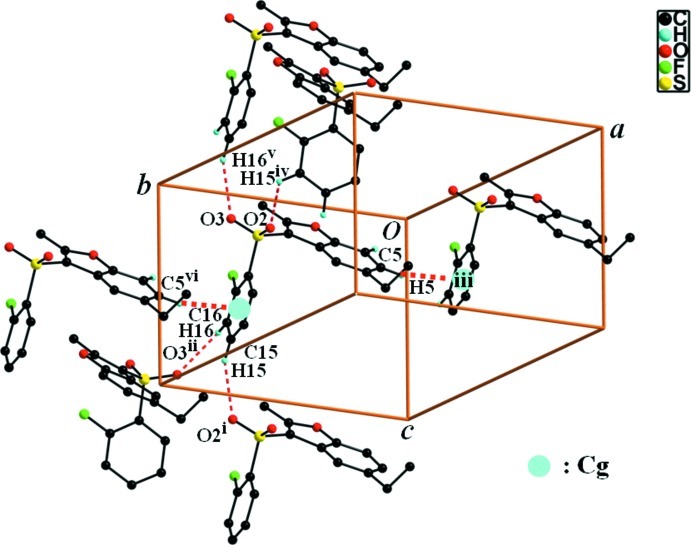
A view of the C—H···O and C—H···π interactions (dotted lines) in the crystal structure of the title compound. H atoms non-participating in hydrogen-bonding were omitted for clarity. [Symmetry codes: (i) x, y, z + 1; (ii) x -1/2, - y + 3/2, z + 1/2; (iii) x + 1/2, y - 1/2, z; (iv) x, y, z - 1; (v) x + 1/2, - y + 3/2, z - 1/2; (vi) x - 1/2, y + 1/2, z.]

## supplementary crystallographic information

### Comment

As a part of our ongoing study of 5-ethyl-2-methyl-1-benzofuran derivatives containing 3-(4-fluorophenylsulfonyl) (Choi *et al.* , 2010) and 3-(3-fluorophenylsulfonyl) (Choi *et al.* , 2011) substituents, we report herein the crystal structure of the title compound.

The title compound crystallizes as the non-centrosymmetric space group *C*_c_ in spite of having no asymmetric C atoms.

In the title molecule (Fig. 1), the benzofuran unit is essentially planar, with a mean deviation of 0.016 (2) Å from the least-squares plane defined by the nine constituent atoms. The dihedral angle between the 2-fluorophenyl ring and the mean plane of the benzofuran fragment is 89.12 (8)°. In the crystal structure (Fig. 2), molecules are connected by weak C—H···O and C—H···π interactions (Table 1, Cg is the centroid of the C12-C17 2-fluorophenyl ring).

### Experimental

3-Chloroperoxybenzoic acid (77%, 515 mg, 2.3 mmol) was added in small portions to a stirred solution of 5-ethyl-3-(2-fluorophenylsulfanyl)-2-methyl-1-benzofuran (315 mg, 1.1 mmol) in dichloromethane (50 mL) at 273 K. After being stirred at room temperature for 10h, the mixture was washed with saturated sodium bicarbonate solution and the organic layer was separated, dried over magnesium sulfate, filtered and concentrated at reduced pressure. The residue was purified by column chromatography (benzene) to afford the title compound as a colorless solid [yield 72%, m.p. 410–411 K; *R*_f_ = 0.61 (benzene)]. Single crystals suitable for X-ray diffraction were prepared by slow evaporation of a solution of the title compound in ethyl acetate at room temperature.

### Refinement

All H atoms were geometrically positioned and refined using a riding model, with C–H = 0.95 Å for aryl, 0.99 Å for the methylene, and 0.98 Å for methyl H atoms. *U*_iso_(H) = 1.2*U*_eq_(C) for aryl and methylene, and 1.5*U*_eq_(C) for methyl H atoms. The positions of methyl hydrogens were optimized rotationally.

### Crystal data

**Table:**

C_17_H_15_FO_3_S	*F*(000) = 664
*M**_r_* = 318.35	*D*_x_ = 1.401 Mg m^−^^3^
Monoclinic, *C**c*	Melting point = 410–411 K
Hall symbol: C -2yc	Mo *K*α radiation, λ = 0.71073 Å
*a* = 11.290 (2) Å	Cell parameters from 2955 reflections
*b* = 16.171 (3) Å	θ = 2.3–27.5°
*c* = 8.5612 (14) Å	µ = 0.24 mm^−^^1^
β = 105.045 (11)°	*T* = 173 K
*V* = 1509.4 (5) Å^3^	Block, colourless
*Z* = 4	0.38 × 0.30 × 0.27 mm

### Data collection

**Table:**

Bruker SMART APEXII CCD diffractometer	3129 independent reflections
Radiation source: rotating anode	2676 reflections with *I* > 2σ(*I*)
Graphite multilayer monochromator	*R*_int_ = 0.035
Detector resolution: 10.0 pixels mm^-1^	θ_max_ = 28.2°, θ_min_ = 2.3°
φ and ω scans	*h* = −13→14
Absorption correction: multi-scan (*SADABS*; Bruker, 2009)	*k* = −21→19
*T*_min_ = 0.545, *T*_max_ = 0.746	*l* = −11→11
7108 measured reflections	

### Refinement

**Table:**

Refinement on *F*^2^	Secondary atom site location: difference Fourier map
Least-squares matrix: full	Hydrogen site location: difference Fourier map
*R*[*F*^2^ > 2σ(*F*^2^)] = 0.042	H-atom parameters constrained
*wR*(*F*^2^) = 0.105	*w* = 1/[σ^2^(*F*_o_^2^) + (0.0518*P*)^2^ + 0.5589*P*] where *P* = (*F*_o_^2^ + 2*F*_c_^2^)/3
*S* = 1.06	(Δ/σ)_max_ < 0.001
3129 reflections	Δρ_max_ = 0.61 e Å^−^^3^
201 parameters	Δρ_min_ = −0.35 e Å^−^^3^
2 restraints	Absolute structure: Flack (1983), 1271 Friedel pairs
Primary atom site location: structure-invariant direct methods	Flack parameter: −0.05 (8)

### Special details

**Table:**

Geometry. All esds (except the esd in the dihedral angle between two l.s. planes) are estimated using the full covariance matrix. The cell esds are taken into account individually in the estimation of esds in distances, angles and torsion angles; correlations between esds in cell parameters are only used when they are defined by crystal symmetry. An approximate (isotropic) treatment of cell esds is used for estimating esds involving l.s. planes.
Refinement. Refinement of F^2^ against ALL reflections. The weighted R-factor wR and goodness of fit S are based on F^2^, conventional R-factors R are based on F, with F set to zero for negative F^2^. The threshold expression of F^2^ > 2sigma(F^2^) is used only for calculating R-factors(gt) etc. and is not relevant to the choice of reflections for refinement. R-factors based on F^2^ are statistically about twice as large as those based on F, and R- factors based on ALL data will be even larger.

### Fractional atomic coordinates and isotropic or equivalent isotropic displacement parameters (Å^2^)

**Table:**

	*x*	*y*	*z*	*U*_iso_*/*U*_eq_	
S1	0.14686 (6)	0.71642 (4)	0.27469 (7)	0.03361 (16)	
O1	0.13896 (19)	0.47875 (12)	0.1852 (2)	0.0427 (5)	
O2	0.2615 (2)	0.75833 (13)	0.2964 (3)	0.0462 (5)	
O3	0.0429 (2)	0.74224 (14)	0.1475 (2)	0.0503 (6)	
F1	−0.07139 (15)	0.64592 (12)	0.3612 (2)	0.0498 (4)	
C1	0.1696 (2)	0.61168 (16)	0.2571 (3)	0.0316 (5)	
C2	0.2695 (2)	0.56448 (15)	0.3603 (3)	0.0297 (5)	
C3	0.3736 (2)	0.58219 (17)	0.4846 (3)	0.0331 (5)	
H3	0.3936	0.6377	0.5176	0.040*	
C4	0.4472 (3)	0.51754 (17)	0.5588 (3)	0.0366 (6)	
C5	0.4155 (3)	0.43570 (18)	0.5073 (4)	0.0455 (7)	
H5	0.4655	0.3916	0.5608	0.055*	
C6	0.3147 (3)	0.41750 (18)	0.3825 (4)	0.0456 (7)	
H6	0.2949	0.3623	0.3474	0.055*	
C7	0.2437 (3)	0.48350 (17)	0.3110 (4)	0.0364 (6)	
C8	0.0946 (2)	0.55781 (18)	0.1554 (3)	0.0375 (6)	
C9	0.5622 (3)	0.5333 (2)	0.6926 (3)	0.0463 (7)	
H9A	0.5547	0.5875	0.7426	0.056*	
H9B	0.5690	0.4904	0.7770	0.056*	
C10	0.6777 (3)	0.5328 (3)	0.6354 (5)	0.0625 (10)	
H10A	0.6869	0.4788	0.5880	0.094*	
H10B	0.7485	0.5433	0.7272	0.094*	
H10C	0.6726	0.5760	0.5537	0.094*	
C11	−0.0195 (3)	0.5648 (2)	0.0229 (4)	0.0544 (8)	
H11A	−0.0508	0.6215	0.0184	0.082*	
H11B	−0.0812	0.5264	0.0429	0.082*	
H11C	−0.0019	0.5510	−0.0803	0.082*	
C12	0.1073 (2)	0.72538 (15)	0.4600 (3)	0.0278 (5)	
C13	0.1835 (2)	0.76928 (16)	0.5882 (3)	0.0350 (6)	
H13	0.2585	0.7921	0.5772	0.042*	
C14	0.1496 (3)	0.77939 (18)	0.7304 (3)	0.0421 (7)	
H14	0.2010	0.8096	0.8171	0.051*	
C15	0.0410 (3)	0.7457 (2)	0.7474 (3)	0.0427 (7)	
H15	0.0180	0.7532	0.8456	0.051*	
C16	−0.0339 (3)	0.70139 (18)	0.6230 (4)	0.0402 (6)	
H16	−0.1082	0.6778	0.6347	0.048*	
C17	0.0007 (2)	0.69186 (17)	0.4819 (3)	0.0335 (5)	

### Atomic displacement parameters (Å^2^)

**Table:**

	*U*^11^	*U*^22^	*U*^33^	*U*^12^	*U*^13^	*U*^23^
S1	0.0419 (3)	0.0300 (3)	0.0335 (3)	0.0059 (3)	0.0181 (2)	0.0064 (3)
O1	0.0395 (11)	0.0359 (11)	0.0531 (11)	−0.0064 (9)	0.0129 (9)	−0.0118 (9)
O2	0.0523 (12)	0.0343 (11)	0.0632 (13)	−0.0018 (9)	0.0352 (11)	0.0056 (9)
O3	0.0683 (16)	0.0478 (13)	0.0349 (10)	0.0197 (11)	0.0135 (10)	0.0118 (9)
F1	0.0375 (9)	0.0586 (11)	0.0550 (9)	−0.0101 (8)	0.0147 (8)	−0.0151 (8)
C1	0.0346 (14)	0.0315 (12)	0.0326 (12)	0.0013 (10)	0.0158 (10)	−0.0010 (10)
C2	0.0329 (12)	0.0235 (12)	0.0368 (12)	0.0006 (10)	0.0162 (10)	−0.0004 (9)
C3	0.0374 (14)	0.0284 (13)	0.0360 (12)	0.0005 (11)	0.0141 (11)	−0.0004 (10)
C4	0.0390 (15)	0.0349 (14)	0.0379 (13)	0.0045 (11)	0.0137 (11)	0.0038 (11)
C5	0.0484 (17)	0.0307 (15)	0.0598 (18)	0.0084 (13)	0.0181 (14)	0.0088 (13)
C6	0.0481 (17)	0.0252 (15)	0.0667 (19)	−0.0008 (12)	0.0206 (15)	−0.0022 (12)
C7	0.0339 (14)	0.0309 (13)	0.0478 (14)	−0.0030 (11)	0.0170 (11)	−0.0043 (11)
C8	0.0373 (14)	0.0402 (16)	0.0371 (13)	0.0011 (11)	0.0137 (11)	−0.0037 (11)
C9	0.0480 (17)	0.0489 (19)	0.0385 (14)	0.0091 (14)	0.0051 (13)	0.0054 (13)
C10	0.0399 (18)	0.084 (3)	0.058 (2)	−0.0033 (17)	0.0042 (15)	−0.0094 (18)
C11	0.0429 (18)	0.068 (2)	0.0491 (17)	0.0014 (16)	0.0051 (14)	−0.0069 (15)
C12	0.0321 (12)	0.0238 (12)	0.0297 (11)	0.0062 (10)	0.0120 (9)	0.0045 (8)
C13	0.0311 (13)	0.0289 (13)	0.0444 (14)	0.0033 (10)	0.0087 (10)	−0.0004 (10)
C14	0.0450 (16)	0.0420 (16)	0.0360 (14)	0.0112 (13)	0.0044 (13)	−0.0033 (11)
C15	0.0531 (17)	0.0437 (16)	0.0353 (13)	0.0148 (14)	0.0186 (12)	0.0055 (12)
C16	0.0421 (15)	0.0374 (15)	0.0480 (15)	0.0073 (12)	0.0241 (13)	0.0078 (11)
C17	0.0308 (13)	0.0324 (13)	0.0376 (13)	0.0035 (10)	0.0094 (10)	−0.0002 (10)

### Geometric parameters (Å, º)

**Table:**

S1—O2	1.430 (2)	C9—C10	1.507 (5)
S1—O3	1.440 (2)	C9—H9A	0.9900
S1—C1	1.726 (3)	C9—H9B	0.9900
S1—C12	1.762 (2)	C10—H10A	0.9800
O1—C8	1.373 (4)	C10—H10B	0.9800
O1—C7	1.379 (3)	C10—H10C	0.9800
F1—C17	1.359 (3)	C11—H11A	0.9800
C1—C8	1.361 (4)	C11—H11B	0.9800
C1—C2	1.455 (4)	C11—H11C	0.9800
C2—C7	1.384 (4)	C12—C17	1.377 (4)
C2—C3	1.395 (3)	C12—C13	1.400 (3)
C3—C4	1.383 (4)	C13—C14	1.377 (4)
C3—H3	0.9500	C13—H13	0.9500
C4—C5	1.411 (4)	C14—C15	1.383 (4)
C4—C9	1.513 (4)	C14—H14	0.9500
C5—C6	1.376 (4)	C15—C16	1.377 (5)
C5—H5	0.9500	C15—H15	0.9500
C6—C7	1.379 (4)	C16—C17	1.371 (4)
C6—H6	0.9500	C16—H16	0.9500
C8—C11	1.484 (4)		
			
O2—S1—O3	119.56 (14)	C10—C9—H9B	108.9
O2—S1—C1	109.03 (12)	C4—C9—H9B	108.9
O3—S1—C1	109.23 (13)	H9A—C9—H9B	107.7
O2—S1—C12	106.06 (13)	C9—C10—H10A	109.5
O3—S1—C12	108.04 (12)	C9—C10—H10B	109.5
C1—S1—C12	103.74 (11)	H10A—C10—H10B	109.5
C8—O1—C7	106.9 (2)	C9—C10—H10C	109.5
C8—C1—C2	108.0 (2)	H10A—C10—H10C	109.5
C8—C1—S1	127.0 (2)	H10B—C10—H10C	109.5
C2—C1—S1	124.86 (18)	C8—C11—H11A	109.5
C7—C2—C3	119.7 (2)	C8—C11—H11B	109.5
C7—C2—C1	104.0 (2)	H11A—C11—H11B	109.5
C3—C2—C1	136.2 (2)	C8—C11—H11C	109.5
C4—C3—C2	118.8 (2)	H11A—C11—H11C	109.5
C4—C3—H3	120.6	H11B—C11—H11C	109.5
C2—C3—H3	120.6	C17—C12—C13	118.3 (2)
C3—C4—C5	119.4 (3)	C17—C12—S1	121.71 (19)
C3—C4—C9	121.0 (3)	C13—C12—S1	120.02 (19)
C5—C4—C9	119.5 (2)	C14—C13—C12	119.9 (3)
C6—C5—C4	122.3 (3)	C14—C13—H13	120.0
C6—C5—H5	118.8	C12—C13—H13	120.0
C4—C5—H5	118.8	C13—C14—C15	120.2 (3)
C5—C6—C7	116.6 (3)	C13—C14—H14	119.9
C5—C6—H6	121.7	C15—C14—H14	119.9
C7—C6—H6	121.7	C16—C15—C14	120.5 (3)
C6—C7—O1	125.8 (3)	C16—C15—H15	119.8
C6—C7—C2	123.0 (3)	C14—C15—H15	119.8
O1—C7—C2	111.1 (2)	C17—C16—C15	118.8 (3)
C1—C8—O1	109.9 (2)	C17—C16—H16	120.6
C1—C8—C11	135.5 (3)	C15—C16—H16	120.6
O1—C8—C11	114.5 (3)	F1—C17—C16	118.7 (2)
C10—C9—C4	113.4 (3)	F1—C17—C12	118.9 (2)
C10—C9—H9A	108.9	C16—C17—C12	122.4 (2)
C4—C9—H9A	108.9		
			
O2—S1—C1—C8	−140.5 (2)	C2—C1—C8—O1	−0.1 (3)
O3—S1—C1—C8	−8.2 (3)	S1—C1—C8—O1	−175.45 (18)
C12—S1—C1—C8	106.8 (2)	C2—C1—C8—C11	−179.2 (3)
O2—S1—C1—C2	44.9 (2)	S1—C1—C8—C11	5.5 (5)
O3—S1—C1—C2	177.2 (2)	C7—O1—C8—C1	1.0 (3)
C12—S1—C1—C2	−67.8 (2)	C7—O1—C8—C11	−179.7 (2)
C8—C1—C2—C7	−0.8 (3)	C3—C4—C9—C10	−98.5 (4)
S1—C1—C2—C7	174.68 (19)	C5—C4—C9—C10	80.6 (4)
C8—C1—C2—C3	179.4 (3)	O2—S1—C12—C17	179.8 (2)
S1—C1—C2—C3	−5.1 (4)	O3—S1—C12—C17	50.5 (2)
C7—C2—C3—C4	−1.7 (4)	C1—S1—C12—C17	−65.4 (2)
C1—C2—C3—C4	178.1 (3)	O2—S1—C12—C13	1.4 (2)
C2—C3—C4—C5	0.0 (4)	O3—S1—C12—C13	−127.9 (2)
C2—C3—C4—C9	179.1 (2)	C1—S1—C12—C13	116.3 (2)
C3—C4—C5—C6	1.5 (5)	C17—C12—C13—C14	−1.3 (3)
C9—C4—C5—C6	−177.6 (3)	S1—C12—C13—C14	177.1 (2)
C4—C5—C6—C7	−1.2 (5)	C12—C13—C14—C15	0.5 (4)
C5—C6—C7—O1	−179.6 (3)	C13—C14—C15—C16	0.4 (4)
C5—C6—C7—C2	−0.5 (5)	C14—C15—C16—C17	−0.5 (4)
C8—O1—C7—C6	177.7 (3)	C15—C16—C17—F1	178.0 (2)
C8—O1—C7—C2	−1.5 (3)	C15—C16—C17—C12	−0.4 (4)
C3—C2—C7—C6	2.0 (4)	C13—C12—C17—F1	−177.1 (2)
C1—C2—C7—C6	−177.8 (3)	S1—C12—C17—F1	4.6 (3)
C3—C2—C7—O1	−178.8 (2)	C13—C12—C17—C16	1.3 (4)
C1—C2—C7—O1	1.4 (3)	S1—C12—C17—C16	−177.1 (2)

### Hydrogen-bond geometry (Å, º)

Cg is the centroid of the C12–C17 2-fluorophenyl ring.

**Table:**

*D*—H···*A*	*D*—H	H···*A*	*D*···*A*	*D*—H···*A*
C15—H15···O3^i^	0.95	2.53	3.420 (3)	156
C16—H16···O2^ii^	0.95	2.49	3.121 (4)	124
C5—H5···*Cg*^iii^	0.95	2.79	3.692 (3)	159

Symmetry codes: (i) *x*, *y*, *z*+1; (ii) *x*−1/2, −*y*+3/2, *z*+1/2; (iii) *x*+1/2, *y*−1/2, *z*.
